# Dopamine D4 Receptor Gene Associated with Fairness Preference in Ultimatum Game

**DOI:** 10.1371/journal.pone.0013765

**Published:** 2010-11-03

**Authors:** Songfa Zhong, Salomon Israel, Idan Shalev, Hong Xue, Richard P. Ebstein, Soo Hong Chew

**Affiliations:** 1 Department of Economics, National University of Singapore, Singapore, Singapore; 2 Scheinfeld Center of Human Genetics for Social Sciences, Hebrew University, Jerusalem, Israel; 3 Applied Genomics Center, Hong Kong University of Science and Technology, Kowloon, Hong Kong; 4 Department of Psychology, National University of Singapore, Singapore, Singapore; 5 Department of Economics, Hong Kong University of Science and Technology, Kowloon, Hong Kong; 6 Department of Finance, National University of Singapore, Singapore, Singapore; 7 Center for Experimental Business Research, Hong Kong University of Science and Technology, Kowloon, Hong Kong; University of Utah, United States of America

## Abstract

In experimental economics, the preference for reciprocal fairness has been observed in the controlled and incentivized laboratory setting of the *ultimatum game*, in which two individuals decide on how to divide a sum of money, with one proposing the share while the second deciding whether to accept. Should the proposal be accepted, the amount is divided accordingly. Otherwise, both would receive no money. A recent twin study has shown that fairness preference inferred from responder behavior is heritable, yet its neurogenetic basis remains unknown. The D4 receptor (DRD4) exon3 is a well-characterized functional polymorphism, which is known to be associated with attention deficit hyperactivity disorder and personality traits including novelty seeking and self-report altruism. Applying a neurogenetic approach, we find that DRD4 is significantly associated with fairness preference. Additionally, the interaction among this gene, season of birth, and gender is highly significant. This is the first result to link preference for reciprocal fairness to a specific gene and suggests that gene × environment interactions contribute to economic decision making.

## Introduction

Whether human nature is selfish has been the subject of longstanding debates in philosophy, the social sciences, and genetics. In contrast with other social sciences, including anthropology, sociology, and psychology, economics distinguishes itself by giving center stage to the selfishness hypothesis. This is in spite of the pervasiveness of prosocial behavior, e.g., giving to genetically unrelated strangers and favoring equitable social outcomes even at some personal cost.

In experimental economics, the preference for reciprocal fairness has been studied using the ultimatum game (UG) [1] involving a sum of money being divided between two individuals. One proposes the share while the other decides whether to accept. Should the proposed share be accepted, the amount is divided accordingly. Otherwise, both receive no money. The prediction of economic analysis based solely on the selfishness motive is clear; the proposer will make a minimal offer which the responder will always find acceptable. However, the literature on UG behavior reveals proposals to be generally close to 60–40 and that responders tend to reject proposals offering less than 30% of the given amount (see, e.g., [2]).

In this literature, while responder behavior is widely used as a proxy for fairness preference, it is noted that proposer behavior confounds fairness preference and strategic consideration. Besides the possible incidence of fairness preference, a more equitable offer by the first mover may reflect a selfish motive – to prevent a fair minded responder from rejecting a highly inequitable offer in favor of both receiving zero. A recent twin study of UG behavior reported the heritability of responder behavior at 42% [3]. It did not report heritability of proposer behavior citing a lack of variation in proposer behavior. This paper seeks to identify particular genes which may contribute to individual difference in fairness preference observed through UG responder behavior.

A candidate gene that we posit may contribute to ‘fair play’ in the UG is the dopamine D4 receptor (DRD4) gene. The DRD4 is characterized by a highly polymorphic VNTR in exon 3 containing a 48 bp repeat [4]. In Caucasian populations, the most common repeat allele is the 4-repeat allele followed by the 7-repeat allele and 2-repeat allele. In Far Eastern groups, the 7-repeat allele is extremely rare and is ‘displaced’ by the 2-repeat allele as the second most common allele [5]. The more common 4-repeat allele has been identified as the conserved ancestral allele [6], while the 7-repeat was generated by a rare mutational event and the 2-repeat is the product of a single recombination event between the 4-repeat and 7-repeat alleles [5]. In addition, the 2-repeat allele appears to confer a functional ability to inhibit cyclic adenosine monophosphate (cAMP) that is intermediate between the 4-repeat and the 7-repeat allele [7]. This evidence suggests that 2-repeat allele may have similar functionality as the 7-repeat allele. Indeed, there are association studies showing that the 2-repeat allele in non-Caucasian populations is similar in that respect to the 7-repeat allele in attention-deficit hyperactivity disorder (ADHD) and other relevant phenotypes and that it is dissimilar to the 4 allele [8], [9], [10], [11], [12].

The DRD4 48 bp VNTR is known for contributing to individual differences in traits including novelty seeking [13], financial risk taking [14], [15], self-report altruism [16], ADHD [17], mood [9], and substance abuse [18]. Beyond association with personality traits, researchers have explored brain mechanisms underlying the links between gene and behavior using imaging genetics, and show that DRD4 modulates brain activations in the right ventrolateral prefrontal cortex and right insula [19], which have further been shown to correlate with UG responder behavior [20], [21]. Additionally, high levels of DRD4 immunoreactivity were observed in the rat insula among other cortical areas [22]. These anatomical studies suggest that the DRD4 plays a major role in mediating cortical dopamine neurotransmission. Interestingly, the DRD4 48 bp VNTR is also associated with cortical thinning in areas important in attentional control suggesting a developmental role for this receptor [23]. The cumulative evidence suggests that DRD4 48 bp VNTR as a candidate for modulating fairness preference.

Complex traits such as sense of fairness are neither the exclusive result of hard wiring, nor shaped entirely by non-genetic factors, but reflect an intricate interplay between genes and environmental elements (‘nature and nurture’). Two seminal studies by Caspi and his colleagues have underscored the importance of gene × environment interactions in violence [24] and depression [25]. A recent study [26] finds evidence for a gene by environment interaction, such that individuals with the low activity form of monoamine oxidase A (MAOA) proved more likely to administer hot sauce as punishment to their opponent when 80% of their earnings were taken in a simple economic power-to-take game than those with the more active version of the gene. There is accumulating evidence that the impact of the DRD4 48 bp VNTR polymorphism on behavior is also fine tuned by environmental inputs. For example, maternal insensitivity was associated with externalizing (oppositional, aggressive) behaviors, but only in the presence of the DRD4 48 bp VNTR 7-repeat allele polymorphism [27]. The increase in externalizing behaviors in children with the 7-repeat allele, and exposed to insensitive care, was six times higher than is the case for children without these combined risks. It is reported that body mass index was higher in those with 7-repeat alleles in the nomadic, but lower among recently settled Ariaal men of northern Kenya [28]. Additionally environmental factors such as season of birth (SoB) have also been demonstrated to balance the impact of the DRD4 48 bp VNTR polymorphism. An interaction has been reported between SoB and the expression of the DRD4 48 bp VNTR in children with hyperkinetic disorder comorbid with conduct disorder as well as in controls, which differ significantly from each other [29]. Non-winter born children carrying the DRD4 48 bp VNTR 7-repeat allele showed higher levels of susceptibility to risk of developing hyperkinetic disorder and conduct disorder. Non-winter born subjects carrying the 7-repeat have higher scores of venturesomeness [30]. Interestingly, the 7-repeat allele is the ‘risk allele’ that interacts with SoB to confer vulnerability to less adaptive behaviors. It is hypothesized [29] that the mutually inhibitory dopamine–melatonin systems are subject to seasonal changes such as temperature allowing season-of-birth and variations of the candidate genes to interact across different dopamine levels during gestation. Additionally, SoB is thought to constitute an overall environmental challenge to the developing fetus with individuals carrying ‘risk alleles’ displaying greater vulnerability to maladaptive behaviors.

## Methods

### Ethics Statement

Each subject gave informed written consent for participation both in the economic experiment and in having his/her blood sample taken. The study including the use of subject payment incentive in the economic experiment and collection of blood sample was approved by the Institutional Review Board at the Hong Kong University of Science and Technology.

### Subjects

We recruited 227 Chinese subjects to participate in the experiment and made use of a self-report questionnaire which includes a question – “Ethnicity: ___” – at the end of the experiment to arrive at 208 Han and 19 non-Han subjects To avoid the conundrum of population stratification, we included only Han Chinese in the analysis of this paper. The demographics information of the subjects is summarized in Table S1.

### Economic experiment

We adhere to the practice in experimental economics of incentivized choice without using deception. Pairs of subjects participate in the UG to divide Y20 (about US$3). In the first stage, each subject plays the role of the first mover and makes a proposed offer to a randomly matched second mover. In the second stage, each subject plays the role of the second mover and states a minimum acceptable offer being the amount below which the responder will reject the offer from a randomly matched first mover [2]. Both paired subjects receive the proposed amounts if proposer's offer exceeds responder's minimum acceptable offer. Otherwise, both receive zero. The entire sample of subjects consists of Han Chinese students born in the Northern Hemisphere. Consistent with standard practice in SoB studies [30], we classify those born between October and March as winter-born, and the others as non-winter born.

### Genotyping

The DRD4 48 bp VNTR was assayed by polymerase chain reaction (PCR) using the primers and reaction conditions, as described in [16]. The polymorphism for the DRD4 48 bp VNTR is characterized using PCR amplification procedure with the following primer: F5′ - CTT CCT ACC CTG CCC GCT CAT GCT GCT GCT CTA CTGG - 3′ and R5′ - ACC ACC ACC GGC AGG ACC CTC ATG GCC TTG CGC TC – 3. PCR reactions were performed using 5 µl Master Mix (Thermo scientific), 2 µl primers (0.5 µM), 0.6 µl Mg/Cl2 (2.5 mM), 0.4 µl DMSO 5% and 1 µl of water to total of 9 µl total volume and an additional 1 µl of genomic DNA was added to the mixture. All PCR reactions were employed on a Biometra T1 Thermocycler (Biometra, Güttingem, Germany). PCR reaction condition is as follows: preheating step at 94.0 °C for 5 min, 34 cycles of denaturation at 94.0 °C for 30 s, reannealing at 55 °C for 30 s, and extension at 72 °C for 90 s. The reaction proceeded to a hold at 72 °C for 5 min. The reaction mixture was then electrophoresed on a 3% agarose gel (AMRESCO) with ethidium bromide to screen for genotypes.

The distribution of genotype frequency – 4/4 (55.5%), 2/4 (34.0%), 4/5 (4.5%), 2/2 (3.5%), 4/3 (3.5%), 4/7 (2.0%), and 2/3 (0.5%) – is comparable with other studies with Chinese population [5], [6], and is in Hardy-Weinberg equilibrium (Chi-square test, p = 0.383). Reist et al. [8] have suggested that the 7-repeat allele and 2-repeat allele are similar functionally and distinct from the 4-repeat allele, the ancestral allele [5], [6]. The 2-repeat allele was associated with novelty seeking traits in some investigations involving Asian populations [9], similar to what has been often observed for the 7-repeat allele [13]. Here subjects were grouped into two categories, 2/2 & 2/4 versus 4/4, excluding twenty two subjects with 2/3, 3/4, 4/5, and 4/7. Our results are robust to inclusion of these genotypes.

### Statistical Analysis

In this study, we focus on testing the effect of DRD4 48 bp VNTR and its interactions with SoB and gender. In association studies, multiple testing needs to be considered when multiple markers are tested independently on a phenotype [31], [32]. In our case, multiple testing correction is not needed since we use an omnibus test with a regression model involving DRD4 48 bp VNTR, SoB, gender and their interaction terms. As with association studies in general, the current investigation should be considered provisional until replicated in independent samples. We use simple linear regression with robust standard error in Stata 10 (Dataset S1).

## Results

The proposer's average offer is 9.09 (45.5%) out of Y20, while the responder's average minimum acceptable offer is Y6.05 (30.3%) out of Y20. This is similar to the results of other UG experiments [2]. The summary statistics by genotype, SoB, and gender are presented in Table 1. Male subjects state a significantly higher minimal acceptable offer than female subjects (t-test, p = 0.030). This is consistent with previous findings [33], [34] with the exception of a 89-subject study [35], which did not find gender difference. Clearly, further studies are required to resolve the question of whether there is gender difference in UG behavior. Notably, the DRD4 48 bp VNTR has a significant effect on responder behavior (t-test, p = 0.010), where subjects with the 4/4 genotype state a 25.6% higher minimal acceptable offer than subjects with 2/4 & 2/2 genotypes.

**Table 1:** Summary statistics.

		Responder		Proposer	
Variable	# of Obs	Mean	Std. Dev.	Mean	Std. Dev.
2/2	7	6.14	3.53	8.28	2.36
2/4	67	5.22**	3.37	9.17	2.38
4/4	111	6.67**	3.58	8.93	2.72
others	22	5.41	3.38	9.95	1.79
Male	95	6.63*	3.36	9.17	2.61
Female	113	5.57*	3.62	9.04	2.46
Winter	108	6.12	3.35	8.89	2.43
Non-winter	98	5.98	3.75	9.36	2.59

Univariate regression analysis is performed for DRD4, gender, and SoB. The coefficient is statistically significant either at the ***0.1% level, at the **1% level, or at the *5% level, using two-sided t-tests.

**Figure 1:**
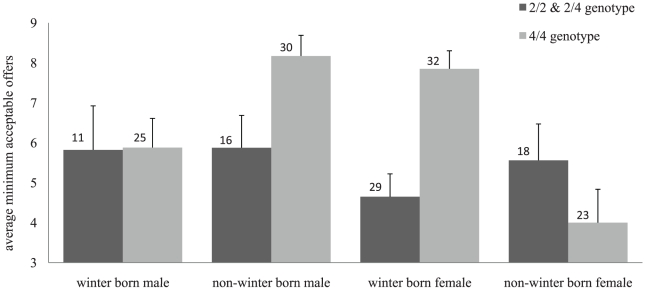
Interaction among DRD4, SoB and Gender. The columns represent the means of minimum acceptable offers of the different groups. Error bars represent standard errors of the means. The number in each column represents the number of subjects in each group. Subjects with 4/4 genotype state a significantly higher minimum acceptable offer than 2/2 & 2/4 genotype for non-winter born males and winter born females.

Figure 1 illustrates the effect of DRD4 48 bp VNTR and its interaction with SoB and gender. Non-winter born male and winter-born female subjects with the 4/4 genotype tend to have a higher minimum acceptable offer than subjects with 2/2 & 2/4 genotype. For winter-born male subjects, DRD4 48 bp VNTR has no effect. For non-winter-born female subjects the effect tends to be opposite, i.e, those with the 2/2 & 2/4 genotype tend to have a higher minimum acceptable offer than subjects with 4/4 genotype.

Table 2 summarizes the effect of DRD4 48 bp VNTR, and its interactions with SoB and gender. The joint effect of the DRD4 48 bp VNTR related regressors in the model (DRD4, DRD4 × SoB, DRD4 × gender, and DRD4 × SoB × gender) is highly significant (F-test: p = 0.0001), supporting the role of DRD4 48 bp VNTR in individual difference in fairness preference. The effect of DRD4 48 bp VNTR is largely captured by interaction terms, and in particular the three way interaction, DRD4 × SoB × gender, is highly significant (t-test: p = 0.001). The presence of the interaction terms, relative to the model without interaction terms, leads to an increase in the adjusted R-squared from 3.6% to 13.7%.This suggests the importance of the interaction among DRD4 48 bp VNTR, SoB, and gender in modulating individual difference in ultimatum game responder's behavior.

**Table 2:** Statistical Results.

Regressor	Model 1	Model 2	Model 3
DRD4	1.358 (0.519)**	1.255 (0.526) *	0.062 (1.302)
SoB		−0.221 (0.548)	0.057 (1.341)
Gender		−0.908(0.552)	−1.163 (1.219)
DRD4 × SoB			2.230 (1.616)
DRD4 × Gender			3.130 (1.494)*
SoB × Gender			0.844 (1.718)
DRD4 × SoB × Gender			−6.973 (2.161)***
Intercept	5.311 (0.392)***	5.990 (0.627) ***	5.818 (1.075)***
Adjusted R-squared	2.9%	3.6%	13.7%

UG responders' minimum acceptable offers are regressed on DRD4 exon3 (2/2 & 2/4 genotype  = 0, 4/4 genotype  = 1), SoB (winter born  = 0; non-winter born  = 1), and gender (male  = 0, female  = 1), and their interaction terms. The first model contains only DRD4; the second model contains DRD4, SoB and gender; the third model contains DRD4, SoB and gender as well as their interaction terms. The number is the estimated regression coefficients and the one in the bracket is the robust standard errors. The individual coefficient is statistically significant either at the ***0.1% level, at the **1% level, or at the *5% level, using two-sided t-tests.

Further analysis was used to deconstruct the effect of season of birth [36]. One possibility is that subjects born in one season may tend to be older than their classmates due to enrollment date in school (September 1 in China). Being older, they might begin to fulfill different social niches because of their greater body size, intelligence and/or maturity. The appended Table S2 displays the result of the additional analysis using the difference between subjects' birth months and their primary school enrollment date in place of season of birth. The adjusted R-squared is 7.5%. Of the two non-nested models, namely SoB and the difference between subjects' birthdates and their primary school enrollment date, the result of using the J-test [37] favors the former. The SoB model cannot be rejected at p<0.305 while the difference between subjects' birthdates and their primary school enrollment date model is rejected at p<0.001.

Climate including ambient temperature is thought to be an important developmental factor which may underpin the season-of-birth effect [38], [39]. We carried out a temperature-based analysis using temperature in Beijing. The result of this procedure shows that the adjusted R-squared is increased to 15.1% from 13.7% (Table S3). Of the two non-nested models, season of birth and temperature, the result of using the J-test [37] favors the latter. The SoB model is rejected at p<0.031 while the temperature model cannot be rejected at p<0.305. Overall, our findings support the temperature hypothesis towards explaining the SoB effect. These results are reported in Table S3 of the supplementary materials.

Our results are also robust with respect to different specifications. In Table S4, we demonstrate robustness after controlling for demographic variables including age, education, height and weight. Further controls included family income, subject's monthly expenses, and whether they are the single child. Table S5 and Table S6 show the robustness after inclusion of other genotypes 2/3, 3/4, 4/5, and 4/7. By contrast, we do not find significant effect of DRD4 48 bp VNTR, or its interaction with SoB and gender for UG proposer behavior (Table S7).

## Discussion

The development of experimental economics since the 1960's offers a controlled laboratory-based approach to observing human preference in terms of the individual's decision making traits [40]. Recently, altruism has been studied in experimental economics using the dictator game in which the subject decides how much of a given amount would be shared anonymously with another subject who is randomly matched [41], [42]. Combining experimental economics and a classical twin design, Cesarini et al [43] find that altruism observed through the dictator game is heritable. At the same time, Knafo et al. [44] and Israel et al. [45] apply a neurogenetic strategy to study altruism in the dictator game and provide the first evidence for the contribution of specific genes to altruism [46].

Ours is the first investigation of the neurogenetics of preference for reciprocal fairness observed through UG responder behavior in an incentivized economic experiment. We find association between the DRD4 48 bp VNTR polymorphism and responder's preference for fairness. Previous studies find that, relative to 7-repeat allele, subjects with 4-repeat allele of DRD4 48 bp VNTR have a higher score of self-reported altruism [16] and lower tendency to be aggressive [27]. These appear consistent with our finding that subjects with 4-repeat allele are more sensitive to sense of fairness. Our result also complements previous finding that DRD4 48 bp VNTR modulates brain activations in the right ventrolateral prefrontal cortex and right insula [19], which have further been shown to correlate with UG responder behavior [20], [21]. We do not find association between UG proposer behavior and the DRD4 48 bp VNTR. This is attributed to the confounding of selfishness motive and sense of fairness, since an equitable proposal may itself reflect a motive to prevent rejection of an unfair offer by a fair-minded responder.

Preference for fairness measured in ultimatum game could be confounded with aggression [47], [48], [49]. Standard one shot UG could also confound the reward size and preference for fairness [50]. An alternative design [50] by varying both the offer amount and the stake size across trials would be able to control the monetary size and fairness preference. Although DRD4 and novelty seeking have been well studied, meta-analytical review of the association between the DRD4 48 bp VNTR polymorphism and novelty seeking suggests there is no overall association with novelty seeking [51], [52]. It should be noted nevertheless, that meta-analysis supports an association with the promoter-region DRD4 C-521T polymorphism. Interestingly, ADHD has been robustly linked to the DRD4 7 repeat and ADHD adults often exhibit novelty seeking traits [53]. Similarly, meta-analysis [54], [55] have questioned the finding of an interaction effect between 5-HTTLPR and stressful life events on risk of depression. At the same time, the findings of these meta-analyses have led to dissenting views [56]. As with association studies in general [31], the validation of our findings awaits replication in independent samples, particularly with different ethnic groups.

A number of studies have investigated the role of the third cytoplasmic loop, coded by the exon three, in DRD4 function [6]. While the overall results are mixed, it appears that there are differences between the 4-repeat allele and 7-repeat allele with the 4-repeat allele being a more efficient receptor than the 7-repeat allele [7], [57], [58]. Additionally, Reist et al [8] have suggested that the 7-repeat allele and 2-repeat allele are similar functionally and distinct from the 4-repeat allele, the ancestral allele [5], [6]. There is less evidence for this contention with some studies suggesting that the 2-repeat allele is at least or perhaps even more efficient than the 4-repeat allele [7], [59], [60], [61]. Interestingly, studies have shown that the distinction in personality traits between the 4-repeat allele and 2-repeat allele is present not only in Asian population groups [9], but also in some Caucasian populations [62]. Indeed, in some studies, it is possible to distinguish between carriers of the 4-repeat allele and 2-repeat allele across a number of traits. For instance, the 2-repeat allele was associated with novelty seeking traits in some investigations involving Asian populations [9], similar to what has been often observed for the 7-repeat allele [13].

In the current paper, subjects carrying the 4-repeat allele display a more exacting benchmark for fairness and demand a more equitable wealth distribution in the UG. The sense of fairness associated with the 4-repeat allele is consistent with its reported role in altruism assessed using a self-report questionnaire [16]. Across human populations, the 4-repeat allele goes along with greater adherence to social norms as opposed to the role of the 7-repeat allele which predisposes the individual to gambling [63], addiction [64], impulsivity [65] and increased sexual drive [66], [67].

The molecular mechanism by which gene × environment interactions being put into place often begins in utero and such fetal programming can have long-term consequences extending into adult life. For instance, SoB has been associated with a wide range of behavioral traits, including suicide [68], [69], schizoid-like features in non-clinical groups [70], impulsivity and sensation seeking [65], novelty seeking [71], self-mutilating behavior [72], schizophrenia [73] and eating attitude [74]. The mechanisms by which SoB impacts behavior are likely to be varied but presumably modification of the epigenome during fetal development is a key pathway that may generate untoward consequences later in life. Adverse long-term behavioral effects likely reflect a mismatch between early (fetal and neonatal) environmental conditions and the conditions that the individual will confront later in life [75]. Since SoB involves a multitude of variables from length of day, rainfall, temperature, variety of foods consumed and many others, it is not a simple task to identify the precise causal factors that are indexed by SoB on behavior. We suggest, however, that one future strategy towards unraveling the mechanisms by which SoB impacts behavior might be assessment of epigenetic changes in CpG methylation patterns overall (e.g. line-1[76]) and at specific genes such as the glucocorticoid receptor ([77], [78]. We suggest the notion that the percent methylation at CpG sites can be operationalized to represent a reliable measure of SoB effects on behavior and it offers an overall proxy for all those variables that are difficult to ascertain reliably otherwise. After all, to affect future behavior SoB needs to ultimately modulate gene expression in the adult brain and changes in the early epigenome apparently do just that [79], [80], [81], [82], [83], [84]. The DRD4 48 bp VNTR would be a good candidate for mediating environmental influences, as well as gender effects, on behavior since the promoter region is rich in CpG islands [85] which may undergo differential methylation modulated by environmental and hormonal fine tuning. It is noteworthy that dissimilar effects of DRD4 48 bp VNTR in males compared to females has been repeatedly observed for personality traits including extraversion [86],forgiveness [9], and heavy drinking [87]. The DRD4 48 bp VNTR would be a good candidate for mediating environmental influences, as well as gender effects, on behavior since the promoter region is rich in CpG islands [85] which may undergo differential methylation modulated by environmental and hormonal fine tuning. It is noteworthy that dissimilar effects of DRD4 48 bp VNTR in males compared to females has been repeatedly observed for personality traits including extraversion [86], forgiveness [9], and heavy drinking [87].

In the course of human evolution, climate change had posed a persistent survival challenge, even over shorter time horizons. For instance, over the past millennium, specific cold spells in China have been associated with decreased harvests, increased warfare, decreased population and dynastic changes [88], [89]. Our finding suggests that the preference for reciprocal fairness is hardwired by common polymorphisms and shows sensitivity to environmental change. In hard times, the benchmark for fairness may vary and increase fitness by allowing environment (e.g. harsh winter, low food supplies, winter flu) to reprogram the internal benchmark of fairness nudging the individual to a state of increased fitness in interpersonal relationship and social interaction. On the other hand, culture has also been shown to play an important role in UG responders' behavior. In a study involving 15 small societies, Henrich et al. [90] reported mean offers ranging from 26 percent to 58 percent with rejection rate for low offers of 20 percent or less ranging from zero to 100 percent. Taken together, these results are consistent with the recent gene-culture co-evolutionary models [91], [92] which combine strategies of cooperation and punishment and predict that local learning dynamics leads to different cultural equilibria.

The current study contributes to the emerging literature on the genetics of economic decision making [3], [44]. Employing a neurogenetic strategy enables researchers to observe the behavioral impact of genetic differences, and uncover possible causal factors in human disposition decision making traits [14], [15], [93], [94], [95], [96]. This approach also complements the recent literature on the neuroeconomics of decision making (see, e.g., [97]), including previous studies on the neural basis of altruism [98], [99] and fairness preference [20], [21], [100], [101]. The DRD4 48 bp VNTR was shown to modulate brain activations in the right ventrolateral prefrontal cortex and right insula [19], which in turn correlates with UG responder behavior [21]. It also adds to recent studies on the effects of hormones on ultimatum game behavior. Van den Bergh and Dewitte [102] show that males with lower 2D:4D digit ratios, reflecting a higher degree of exposure to prenatal androgen [103], are more likely to reject an unfair split in neutral contexts, but more likely to accept unfair offers when viewing pictures with sexual cues in UG. Burnham [49] find that men with higher salivary testosterone levels tend to reject low offers, yet testosterone level is not significantly associated with the level of offers in UG. In a recent pharmacological study, Zak et al [48] find average proposals in the UG were significantly lower for men on testosterone compared to those on placebo. At the same time, the rejection threshold is 5% higher for those on testosterone than those on placebo though the difference is not significant. Zethraes et al [104] find no significant effect of estrogen or testosterone treatment in a group of post-menopausal women on any behaviors measured in a series of economic experiments including altruism, reciprocal fairness, trust, trustworthiness, and risk attitudes. However, Eisenegger et al. [47] show that the sublingual administration of testosterone in women causes a substantial increase in proposal in the ultimatum game. A fruitful new avenue to explore is the link between genes, hormones and brain activity with ultimatum game responder behavior using a combined imaging genetics methodology.

## Supporting Information

Dataset S1 The data Set includes the behavior of the proposer and responder, and information about sex, season, temperature, and school
(0.03 MB XLS)

Table S1 Summary Statistics of Demographic Variables. Monthly family income is category measure: less than 2000, between 2000 and 4000, between 4000 and 6000, between 6000 and 8000, between 8000 and 10000, between 10000 and 12000, between 12000 and 14000, between 14000 and 16000, between 16000 and 18000, between 18000 and 20000, above 20000.
(0.03 MB DOC)

Table S2 Statistical Results for using the difference between subjects' birthdates and their primary school enrollment dates. UG responders' minimum acceptable offers are regressed on DRD4 exon3 (2/2 & 2/4 genotype  = 0, 4/4 genotype  = 1), School (difference between subjects' birthdates and their primary school enrollment dates), and gender (male  = 0, female  = 1), and their interaction terms. The first row contains the regressors in the statistical model. The second to the last row contain estimated regression coefficients, robust standard errors, t-value and p-value respectively. The individual coefficient is statistically significant either at the ***0.1% level, at the **1% level, or at the *5% level, using two-sided t-tests. The adjusted R-squared is 7.5%.
(0.04 MB DOC)

Table S3 Statistical Results for using temperature. UG responders' minimum acceptable offers are regressed on DRD4 exon3 (2/2 & 2/4 genotype  = 0, 4/4 genotype  = 1), Temperature (Beijing temperature as a proxy), and gender (male  = 0, female  = 1), and their interaction terms. The first row contains the regressors in the statistical model. The second to the last row contain estimated regression coefficients, robust standard errors, t-value and p-value respectively. The individual coefficient is statistically significant either at the ***0.1% level, at the **1% level, or at the *5% level, using two-sided t-tests. The adjusted R-squared is 15.1%.
(0.04 MB DOC)

Table S4 Statistical Results after further controls for demographic variables in Table S1. UG responders' minimum acceptable offers are regressed on DRD4 exon3 (2/2 & 2/4 genotype  = 0, 4/4 genotype  = 1), SoB (winter born  = 0; non-winter born  = 1), and gender (male  = 0, female  = 1), and their interaction terms. The first row contains the regressors in the statistical model. The second to the last row contain estimated regression coefficients, robust standard errors, t-value and p-value respectively. The individual coefficient is statistically significant either at the ***0.1% level, at the **1% level, or at the *5% level, using two-sided t-tests. The adjusted R-squared is 12.0%.
(0.05 MB DOC)

Table S5 Statistical Results after inclusion minor genotypes into 2/2&2/4 genotype. UG responders' minimum acceptable offers are regressed on DRD4 exon3 (other genotypes  = 0, 4/4 genotype  = 1), SoB (winter born  = 0; non-winter born  = 1), and gender (male  = 0, female  = 1), and their interaction terms. The first row contains the regressors in the statistical model. The second to the last row contain estimated regression coefficients, robust standard errors, t-value and p-value respectively. The individual coefficient is statistically significant either at the ***0.1% level, at the **1% level, or at the *5% level, using two-sided t-tests. The adjusted R-squared is 7.5%. The adjusted R-squared is 12.7%.
(0.04 MB DOC)

Table S6 Statistical Results after inclusion minor genotypes into 4/4 genotype. UG responders' minimum acceptable offers are regressed on DRD4 exon3 (2/2 & 2/4 genotype  = 0, other genotype  = 1), SoB (winter born  = 0; non-winter born  = 1), and gender (male  = 0, female  = 1), and their interaction terms. The first row contains the regressors in the statistical model. The second to the last row contain estimated regression coefficients, robust standard errors, t-value and p-value respectively. The individual coefficient is statistically significant either at the ***0.1% level, at the **1% level, or at the *5% level, using two-sided t-tests. The adjusted R-squared is 12.9%.
(0.04 MB DOC)

Table S7 Statistical Results Proposers' behavior. UG proposers' offers are regressed on DRD4 exon3 (2/2 & 2/4 genotype  = 0, 4/4 genotype  = 1), SoB (winter born  = 0; non-winter born  = 1), and gender (male  = 0, female  = 1), and their interaction terms. The first row contains the regressors in the statistical model. The second to the last row contain estimated regression coefficients, robust standard errors, t-value and p-value respectively. The individual coefficient is statistically significant either at the ***0.1% level, at the **1% level, or at the *5% level, using two-sided t-tests. The adjusted R-squared is 2.4%.
(0.04 MB DOC)
