# Supplementary material for: Dopamine D4 Receptor Gene Associated with Fairness Preference in Ultimatum Game
Source: PLoS One. 2010 Nov 3;5(11):e13765. doi: 10.1371/journal.pone.0013765 (PMC2972208; doi:10.1371/journal.pone.0013765)
Supplement: Table S1 — Summary Statistics of Demographic Variables. Monthly family income is category measure: less than 2000, between 2000 and 4000, between 4000 and 6000, between 6000 and 8000, between 8000 and 10000, between 10000 and 12000, between 12000 and 14000, between 14000 and 16000, between 16000 and 18000, between 18000 and 20000, above 20000. (0.03 MB DOC) [file pone.0013765.s002.doc]

| Variables | Obs | Mean | Std. Dev. |
| --- | --- | --- | --- |
| Age (year) | 208 | 22.50 | 2.36 |
| Gender (male = 0) | 208 | 0.54 | 0.50 |
| Education (undergraduate = 0) | 208 | 0.39 | 0.49 |
| Weight (KG) | 208 | 58.86 | 10.76 |
| Height (CM) | 208 | 166.83 | 7.82 |
| Siblings (0= one child) | 207 | 0.48 | 0.50 |
| Monthly Family Income | 206 | 2.43 | 1.65 |
| Monthly Expense | 207 | 696 .14 | 414.05 |

**Table S1:** *Summary Statistics of Demographic Variables.* Monthly family income is category measure: less than 2000, between 2000 and 4000, between 4000 and 6000, between 6000 and 8000, between 8000 and 10000, between 10000 and 12000, between 12000 and 14000, between 14000 and 16000, between 16000 and 18000, between 18000 and 20000, above 20000.
