# Supplementary material for: Dopamine D4 Receptor Gene Associated with Fairness Preference in Ultimatum Game
Source: PLoS One. 2010 Nov 3;5(11):e13765. doi: 10.1371/journal.pone.0013765 (PMC2972208; doi:10.1371/journal.pone.0013765)
Supplement: Table S2 — Statistical Results for using the difference between subjects' birthdates and their primary school enrollment dates. UG responders' minimum acceptable offers are regressed on DRD4 exon3 (2/2 & 2/4 genotype = 0, 4/4 genotype = 1), School (difference between subjects' birthdates and their primary school enrollment dates), and gender (male = 0, female = 1), and their interaction terms. The first row contains the regressors in the statistical model. The second to the last row contain estimated regression coefficients, robust standard errors, t-value and p-value respectively. The individual coefficient is statistically significant either at the ***0.1% level, at the **1% level, or at the *5% level, using two-sided t-tests. The adjusted R-squared is 7.5%. (0.04 MB DOC) [file pone.0013765.s003.doc]

| **Regressor** | **Coefficient** | **Std Error** | **t-value** | **p-value** |
| --- | --- | --- | --- | --- |
| DRD4 | 2.259 | 1.340 | 1.69 | 0.094 |
| School | -0.091 | 0.164 | -0.55 | 0.58 |
| Gender | 0.226 | 1.755 | 0.13 | 0.897 |
| DRD4 x School | -0.147 | 0.203 | -0.72 | 0.47 |
| DRD4 x Gender | -5.061 | 2.243 | -2.26 | 0.025 |
| School x Gender | -0.114 | 0.235 | -0.49 | 0.626 |
| DRD4 x School x Gender | 0.689 | 0.305 | 2.26 | 0.025 |
| Intercept | 6.335 | 1.106 | 5.73 | 0 |

**Table.S2**. Statistical Results for using the difference between subjects’ birthdates and their primary school enrollment dates

UG responders’ minimum acceptable offers are regressed on DRD4 exon3 (2/2 & 2/4 genotype = 0, 4/4 genotype = 1), School (difference between subjects’ birthdates and their primary school enrollment dates), and gender (male = 0, female = 1), and their interaction terms. The first row contains the regressors in the statistical model. The second to the last row contain estimated regression coefficients, robust standard errors, t-value and p-value respectively. The individual coefficient is statistically significant either at the ***0.1% level, at the **1% level, or at the *5% level, using two-sided t-tests. The adjusted R-squared is 7.5%.
