# Supplementary material for: Dopamine D4 Receptor Gene Associated with Fairness Preference in Ultimatum Game
Source: PLoS One. 2010 Nov 3;5(11):e13765. doi: 10.1371/journal.pone.0013765 (PMC2972208; doi:10.1371/journal.pone.0013765)
Supplement: Table S3 — Statistical Results for using temperature. UG responders' minimum acceptable offers are regressed on DRD4 exon3 (2/2 & 2/4 genotype = 0, 4/4 genotype = 1), Temperature (Beijing temperature as a proxy), and gender (male = 0, female = 1), and their interaction terms. The first row contains the regressors in the statistical model. The second to the last row contain estimated regression coefficients, robust standard errors, t-value and p-value respectively. The individual coefficient is statistically significant either at the ***0.1% level, at the **1% level, or at the *5% level, using two-sided t-tests. The adjusted R-squared is 15.1%. (0.04 MB DOC) [file pone.0013765.s004.doc]

| **Regressor** | **Coefficient** | **Std Error** | **t-value** | **p-value** |
| --- | --- | --- | --- | --- |
| DRD4 | -0.065 | 1.730 | -0.04 | 0.97 |
| temperature | 0.034 | 0.060 | 0.58 | 0.562 |
| Gender | -1.079 | 1.626 | -0.66 | 0.508 |
| DRD4 x temperature | 0.068 | 0.072 | 0.95 | 0.344 |
| DRD4 x Gender | 5.644 | 1.994 | 2.83 | 0.005 |
| Temperature x Gender | 0.028 | 0.076 | 0.38 | 0.706 |
| DRD4 x Temperature x Gender | -0.326 | 0.094 | -3.46 | 0.001 |
| Intercept | 5.136 | 1.423 | 3.61 | 0 |

**Table.S3**. Statistical Results for using temperature.

UG responders’ minimum acceptable offers are regressed on DRD4 exon3 (2/2 & 2/4 genotype = 0, 4/4 genotype = 1), Temperature (Beijing temperature as a proxy), and gender (male = 0, female = 1), and their interaction terms. The first row contains the regressors in the statistical model. The second to the last row contain estimated regression coefficients, robust standard errors, t-value and p-value respectively. The individual coefficient is statistically significant either at the ***0.1% level, at the **1% level, or at the *5% level, using two-sided t-tests. The adjusted R-squared is 15.1%.
