# Supplementary material for: Dopamine D4 Receptor Gene Associated with Fairness Preference in Ultimatum Game
Source: PLoS One. 2010 Nov 3;5(11):e13765. doi: 10.1371/journal.pone.0013765 (PMC2972208; doi:10.1371/journal.pone.0013765)
Supplement: Table S4 — Statistical Results after further controls for demographic variables in Table S1. UG responders' minimum acceptable offers are regressed on DRD4 exon3 (2/2 & 2/4 genotype = 0, 4/4 genotype = 1), SoB (winter born = 0; non-winter born = 1), and gender (male = 0, female = 1), and their interaction terms. The first row contains the regressors in the statistical model. The second to the last row contain estimated regression coefficients, robust standard errors, t-value and p-value respectively. The individual coefficient is statistically significant either at the ***0.1% level, at the **1% level, or at the *5% level, using two-sided t-tests. The adjusted R-squared is 12.0%. (0.05 MB DOC) [file pone.0013765.s005.doc]

| **Regressor** | **Coef.** | **Std. Err.** | **t -value** | **p - value** |
| --- | --- | --- | --- | --- |
| DRD4 | -0.197 | 1.377 | -0.14 | 0.887 |
| Gender | -0.347 | 1.417 | -0.24 | 0.807 |
| SoB | -0.281 | 1.445 | -0.19 | 0.846 |
| DRD4 x SoB | 2.709 | 1.705 | 1.59 | 0.114 |
| DRD4 x Gender | 3.063 | 1.561 | 1.96 | 0.051 |
| SoB x Gender | 1.082 | 1.773 | 0.61 | 0.543 |
| DRD4 x SoB x Gender | -7.044 | 2.190 | -3.22 | 0.002** |
| age | 0.065 | 0.146 | 0.45 | 0.654 |
| Education | 0.631 | 0.702 | 0.9 | 0.369 |
| Weight | 0.068 | 0.056 | 1.22 | 0.226 |
| Height | -0.004 | 0.039 | -0.11 | 0.91 |
| Siblings | 0.361 | 0.522 | 0.69 | 0.49 |
| Family Income | 0.050 | 0.199 | 0.25 | 0.801 |
| Monthly Expense | 0.000 | 0.001 | -0.2 | 0.846 |
| Intercept | -7.359 | 9.494 | -0.78 | 0.439 |

**Table.S4**. *Statistical Results after further controls for demographic variables in Table S1.* UG responders’ minimum acceptable offers are regressed on DRD4 exon3 (2/2 & 2/4 genotype = 0, 4/4 genotype = 1), SoB (winter born = 0; non-winter born = 1), and gender (male = 0, female = 1), and their interaction terms. The first row contains the regressors in the statistical model. The second to the last row contain estimated regression coefficients, robust standard errors, t-value and p-value respectively. The individual coefficient is statistically significant either at the ***0.1% level, at the **1% level, or at the *5% level, using two-sided t-tests. The adjusted R-squared is 12.0%.
