# Supplementary material for: Dopamine D4 Receptor Gene Associated with Fairness Preference in Ultimatum Game
Source: PLoS One. 2010 Nov 3;5(11):e13765. doi: 10.1371/journal.pone.0013765 (PMC2972208; doi:10.1371/journal.pone.0013765)
Supplement: Table S5 — Statistical Results after inclusion minor genotypes into 2/2&2/4 genotype. UG responders' minimum acceptable offers are regressed on DRD4 exon3 (other genotypes = 0, 4/4 genotype = 1), SoB (winter born = 0; non-winter born = 1), and gender (male = 0, female = 1), and their interaction terms. The first row contains the regressors in the statistical model. The second to the last row contain estimated regression coefficients, robust standard errors, t-value and p-value respectively. The individual coefficient is statistically significant either at the ***0.1% level, at the **1% level, or at the *5% level, using two-sided t-tests. The adjusted R-squared is 7.5%. The adjusted R-squared is 12.7%. (0.04 MB DOC) [file pone.0013765.s006.doc]

| **Regressor** | **Coef.** | **Std. Err.** | **t - value** | **p - value** |
| --- | --- | --- | --- | --- |
| DRD4 | 0.056 | 1.128 | 0.05 | 0.960 |
| Sob | 0.224 | 1.078 | 0.21 | 0.836 |
| Gender | -1.000 | 1.012 | -0.99 | 0.324 |
| DRD4 x SoB | 2.063 | 1.404 | 1.47 | 0.143 |
| DRD4 x Gender | 2.967 | 1.329 | 2.23 | 0.027* |
| SoB x Gender | 0.036 | 1.439 | 0.02 | 0.980 |
| DRD4 x SoB x Gender | -6.165 | 1.944 | -3.17 | 0.002** |
| Intercept | 5.824 | 0.858 | 6.79 | 0.000*** |

**Table.S5**. *Statistical Results after inclusion minor genotypes into 2/2&2/4 genotype.* UG responders’ minimum acceptable offers are regressed on DRD4 exon3 (other genotypes = 0, 4/4 genotype = 1), SoB (winter born = 0; non-winter born = 1), and gender (male = 0, female = 1), and their interaction terms. The first row contains the regressors in the statistical model. The second to the last row contain estimated regression coefficients, robust standard errors, t-value and p-value respectively. The individual coefficient is statistically significant either at the ***0.1% level, at the **1% level, or at the *5% level, using two-sided t-tests. The adjusted R-squared is 7.5%. The adjusted R-squared is 12.7%.
