# Supplementary material for: Dopamine D4 Receptor Gene Associated with Fairness Preference in Ultimatum Game
Source: PLoS One. 2010 Nov 3;5(11):e13765. doi: 10.1371/journal.pone.0013765 (PMC2972208; doi:10.1371/journal.pone.0013765)
Supplement: Table S6 — Statistical Results after inclusion minor genotypes into 4/4 genotype. UG responders' minimum acceptable offers are regressed on DRD4 exon3 (2/2 & 2/4 genotype = 0, other genotype = 1), SoB (winter born = 0; non-winter born = 1), and gender (male = 0, female = 1), and their interaction terms. The first row contains the regressors in the statistical model. The second to the last row contain estimated regression coefficients, robust standard errors, t-value and p-value respectively. The individual coefficient is statistically significant either at the ***0.1% level, at the **1% level, or at the *5% level, using two-sided t-tests. The adjusted R-squared is 12.9%. (0.04 MB DOC) [file pone.0013765.s007.doc]

| **Regressor** | **Coef.** | **Std. Err.** | **t -value** | **p - value** |
| --- | --- | --- | --- | --- |
| DRD4 | 0.053 | 1.255 | 0.04 | 0.966 |
| SoB | 0.057 | 1.338 | 0.04 | 0.966 |
| Gender | -1.163 | 1.216 | -0.96 | 0.340 |
| DRD4 x SoB | 2.015 | 1.563 | 1.29 | 0.199 |
| DRD4 x Gender | 2.862 | 1.452 | 1.97 | 0.050* |
| SoB x Gender | 0.844 | 1.714 | 0.49 | 0.623 |
| DRD4 x SoB x Gender | -6.551 | 2.079 | -3.15 | 0.002** |
| Intercept | 5.818 | 1.072 | 5.43 | 0.000*** |

**Table.S6**. *Statistical Results after inclusion minor genotypes into 4/4 genotype.* UG responders’ minimum acceptable offers are regressed on DRD4 exon3 (2/2 & 2/4 genotype = 0, other genotype = 1), SoB (winter born = 0; non-winter born = 1), and gender (male = 0, female = 1), and their interaction terms. The first row contains the regressors in the statistical model. The second to the last row contain estimated regression coefficients, robust standard errors, t-value and p-value respectively. The individual coefficient is statistically significant either at the ***0.1% level, at the **1% level, or at the *5% level, using two-sided t-tests. The adjusted R-squared is 12.9%.
