# Supplementary material for: Dopamine D4 Receptor Gene Associated with Fairness Preference in Ultimatum Game
Source: PLoS One. 2010 Nov 3;5(11):e13765. doi: 10.1371/journal.pone.0013765 (PMC2972208; doi:10.1371/journal.pone.0013765)
Supplement: Table S7 — Statistical Results Proposers' behavior. UG proposers' offers are regressed on DRD4 exon3 (2/2 & 2/4 genotype = 0, 4/4 genotype = 1), SoB (winter born = 0; non-winter born = 1), and gender (male = 0, female = 1), and their interaction terms. The first row contains the regressors in the statistical model. The second to the last row contain estimated regression coefficients, robust standard errors, t-value and p-value respectively. The individual coefficient is statistically significant either at the ***0.1% level, at the **1% level, or at the *5% level, using two-sided t-tests. The adjusted R-squared is 2.4%. (0.04 MB DOC) [file pone.0013765.s008.doc]

| **Regressor** | **Coef.** | **Std. Err.** | **t -value** | **p - value** |
| --- | --- | --- | --- | --- |
| DRD4 | -0.876 | 0.738 | -1.19 | 0.237 |
| SoB | -0.166 | 1.032 | -0.16 | 0.873 |
| Gender | -0.912 | 0.609 | -1.50 | 0.136 |
| DRD4 x SoB | 0.706 | 1.242 | 0.57 | 0.571 |
| DRD4 x Gender | 0.840 | 0.975 | 0.86 | 0.390 |
| SoB x Gender | 0.442 | 1.167 | 0.38 | 0.706 |
| DRD4 x SoB x Gender | -0.539 | 1.575 | -0.34 | 0.733 |
| Intercept | 9.636 | 0.497 | 19.41 | 0.000*** |

**Table.S7**. *Statistical Results Proposers’ behavior.* UG proposers’ offers are regressed on DRD4 exon3 (2/2 & 2/4 genotype = 0, 4/4 genotype = 1), SoB (winter born = 0; non-winter born = 1), and gender (male = 0, female = 1), and their interaction terms. The first row contains the regressors in the statistical model. The second to the last row contain estimated regression coefficients, robust standard errors, t-value and p-value respectively. The individual coefficient is statistically significant either at the ***0.1% level, at the **1% level, or at the *5% level, using two-sided t-tests. The adjusted R-squared is 2.4%.
